# Pulmonary Aspergillosis in Critically Ill COVID-19 Patients Admitted to the Intensive Care Unit: A Retrospective Cohort Study

**DOI:** 10.3390/jof9030315

**Published:** 2023-03-03

**Authors:** Felix Bergmann, Anselm Jorda, Amelie Blaschke, Cornelia Gabler, Serhii Bohdan, Alina Nussbaumer-Pröll, Christine Radtke, Markus Zeitlinger

**Affiliations:** 1 Department of Clinical Pharmacology, Medical University of Vienna, Währinger Gürtel 18-20, 1090 Vienna, Austria; 2 Department of Plastic, Reconstructive and Aesthetic Surgery, Medical University of Vienna, Währinger Gürtel 18-20, 1090 Vienna, Austria; 3 Department of Infectiology and Tropical Medicine, University Clinic of Internal Medicine I, Medical University Vienna, Währinger Gürtel 18-20, 1090 Vienna, Austria; 4 IT Systems and Communications, Medical University of Vienna, Währinger Gürtel 18-20, 1090 Vienna, Austria

**Keywords:** ICU, CAPA, IPA, SARS-CoV-2, co-infection, fungal, aspergillus

## Abstract

COVID-19-associated pulmonary aspergillosis (CAPA) is a life-threatening fungal infection that mainly affects critically ill patients. The aim of this study was to assess the incidence and clinical outcomes of putative CAPA in critically ill COVID-19 patients. This retrospective observational cohort study included 181 cases from 5 ICUs at Vienna General Hospital between January 2020 and April 2022. Patients were diagnosed with putative CAPA according to the AspICU classification, which included a positive Aspergillus culture in a bronchoalveolar lavage sample, compatible signs and symptoms, and abnormal medical imaging. The primary outcome was adjusted 60-day all-cause mortality from ICU admission in patients with vs. without putative CAPA. Secondary outcomes included time from ICU admission to CAPA diagnosis and pathogen prevalence and distribution. Putative CAPA was identified in 35 (19.3%) of 181 COVID-19 patients. The mean time to diagnosis was 9 days. Death at 60 days occurred in 18 of 35 (51.4%) patients with CAPA and in 43 of 146 (29.5%) patients without CAPA (adjusted HR (95%CI) = 2.15 (1.20–3.86, *p* = 0.002). The most frequently isolated Aspergillus species was *Aspergillus fumigatus.* The prevalence of putative pulmonary aspergillosis in critically ill COVID-19 patients was high and was associated with significantly higher mortality.

## 1. Introduction

COVID-19-associated pulmonary aspergillosis (CAPA) is a serious complication associated with respiratory failure caused by species of the genus Aspergillus [1]. The ongoing COVID-19 pandemic has led to a significant increase in the number of patients requiring hospitalization, particularly in intensive care units (ICUs) [2]. Although viral pneumonia has been recognized as the main cause of severity and mortality, COVID-19 has also been linked to other complications such as coagulation disorders, neurological complications, vascular complications, and gastrointestinal and renal disorders [3,4]. Previous studies have shown that critically ill COVID-19 patients are susceptible to other bacterial, viral, and fungal co-infections (within the first 48 h of admission) and secondary infections (≥48 h of admission), including CAPA [5,6,7,8,9]. Damage of the bronchial mucosa and alveolar injury by the virus, in combination with increased pulmonary epithelial and vascular permeability, may create favorable conditions for the invasion of *Aspergillus* spp. [10]. In addition to the direct effects of the virus, the use of glucocorticoids and other immunosuppressive therapies to treat COVID-19 may increase the risk of developing CAPA [11,12].

Despite increasing reports of CAPA, the true prevalence of the complication remains uncertain. Some studies have investigated the prevalence of CAPA, but results have found inconsistent frequencies ranging from 2.5 to 35% [13]. This may be due to differences in the underlying study designs, case definitions, and geographical differences. In addition, the previous literature has reported that the diagnostic performance of common tests for CAPA, such as the galactomannan antigen assay, is limited [14,15]. These heterogeneous results may obscure the true prevalence of invasive pulmonary aspergillosis in COVID-19 patients.

To address these challenges, the present study presents a retrospective analysis of aspergillosis in COVID-19 patients treated in a tertiary care hospital and describes the prevalence, clinical outcomes, and antifungal treatment in this patient population.

## 2. Methods

### 2.1. Study Design and Setting

The present retrospective observational cohort study was conducted at a single European study site (Vienna General Hospital, Vienna, Austria). Prior to the initiation of the study, ethics approval was obtained from the competent Ethics Committee (EC 2259/2021). Data were automatically extracted from medical records according to the inclusion and exclusion criteria. Unavailable data were manually extracted and added to the main data set. The present study was conducted according to the STROBE (Strengthening the Reporting of Observational Studies in Epidemiology) recommendations [16].

### 2.2. Study Population

We included patients admitted to five ICUs with acute respiratory failure and COVID-19, confirmed by polymerase chain reaction (PCR) at the time of hospitalization, with at least one microbiological culture performed in a bronchoalveolar lavage (BAL) sample after ICU admission. All COVID-19 cases were screened from the start of the pandemic through April 2022. We excluded patients < 18 years of age and cases with an ICU duration of ≤48 h.

### 2.3. Definition of Pulmonary Aspergillosis

Patients were diagnosed with putative CAPA according to the AspICU classification by Blot et al. [17]. In brief, criteria included a combination of a positive *Aspergillus* culture in a BAL sample, compatible clinical signs and symptoms of aspergillosis, including worsening respiratory insufficiency despite ventilatory support, abnormal medical imaging in chest X-ray or CT scan, including diffuse reticular or alveolar opacities or nonspecific infiltrates and consolidation, and glucocorticoid treatment (over 20 mg of prednisolone per day or an equivalent dose of any other glucocorticoid). Respiratory sampling was performed at the discretion of the attending physician as part of routine care. Antigen testing for galactomannan from serum and respiratory tract samples was not included in the analysis due to their limited sensitivity, as reported in the previous literature [15].

### 2.4. Outcome Parameters

The primary outcome of this study was the adjusted hazard ratio of 60-day all-cause mortality from ICU admission in patients with or without putative CAPA. Secondary outcomes included the unadjusted hazard ratio of 60-day all-cause mortality from ICU admission, pathogen prevalence and distribution, and time from ICU admission to CAPA diagnosis. In addition, we also assessed the use of immunosuppressants during hospitalization and antifungal therapy after CAPA diagnosis.

### 2.5. Statistical Analysis

Baseline characteristics were reported descriptively using mean ± standard deviation (SD), median (IQR), or numbers (%). Characteristics were compared using an independent t-test for age, weight, height, and body mass index (BMI) or Chi-square test for sex and chronic diseases. The incidence of putative CAPA, microbiological sampling frequencies, and pathogen prevalence and distribution were reported descriptively. Hazard ratios with 95% confidence interval (95% CI) were calculated for the 60-day all-cause mortality. Patients discharged alive were censored at the time of exit. We additionally performed a Cox regression analysis to adjust the hazard ratio for age, sex, BMI, cardiovascular disease, chronic kidney disease, chronic obstructive pulmonary disease, diabetes, cancer diagnosis, and SARS-CoV-2 variant. Statistical analyses and visualizations were performed using R (Version 2021.09.2) and GraphPad Prism 9.3.1.

## 3. Results

### 3.1. Study Population

**Figure 1:**
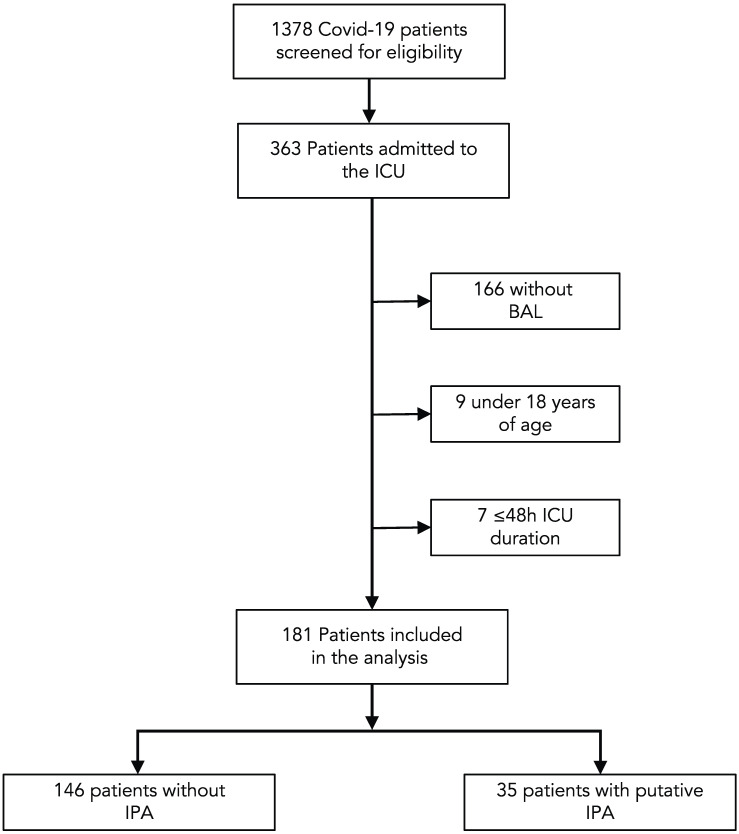
Flow chart of the study population.

In total, 1378 patients with PCR-confirmed COVID-19 infection were identified from January 2020 through April 2022, of which 363 were admitted to an ICU. After excluding 9 cases <18 years of age, 166 patients without microbiological cultures from BAL samples and 7 patients with an ICU duration of ≤48 h, the final analysis set consisted of 181 cases (Figure 1).

The characteristics of the study population are summarized in Table 1. In total, 54 of 181 (29.8%) of patients were female, the overall mean ± SD age was 54 ± 13 years and the mean ± SD BMI was 30.7 ± 7.1. In addition, 133 of 181 (73.5%) patients received immunosuppressants at the time of ICU admission. The mean ± SD duration of intensive care was 36.73 (±25.5) days. The most common comorbidities were obesity (84 of 181 (46.4%)), hypertension (42 of 181 (23.2%)), and mental disorders (33 of 181 (18.2%)). Galactomannan indices and β-D-Glucan levels were higher and interpreted as positive more often in patients with putative CAPA (Table 1).

A total of 35 of 181 (19.3%) cases developed putative CAPA according to the AspICU criteria by Blot et al. [17]. Among these 35 cases, 9 were diagnosed within 48 h of ICU admission, while 26 were diagnosed 48 h or more after admission. The CAPA group had a numerically higher proportion of male patients than non-CAPA cases (74.3% vs. 69.2%). The mean ± SD age was similar between patients with CAPA (56 ± 11 years) and without (54 ± 14 years) CAPA. Co-morbidities were well balanced, with the only significant difference being obesity between the two groups. There was no significant difference in the use of immunosuppressive medication at ICU admission between patients with CAPA (22 of 35 (62.9%)) and without CAPA (110 of 146 (75.3%)).

In total, 34 of 181 (18.8%) of COVID-19 cases were caused by the wildtype virus, 80 (44.2%) by the alpha variant, and 54 (29.8%) by the delta variant. Thirteen (7.2%) cases were not sequenced. There was no significant difference in underlying variants between groups.

### 3.2. Mortality

**Figure 2:**
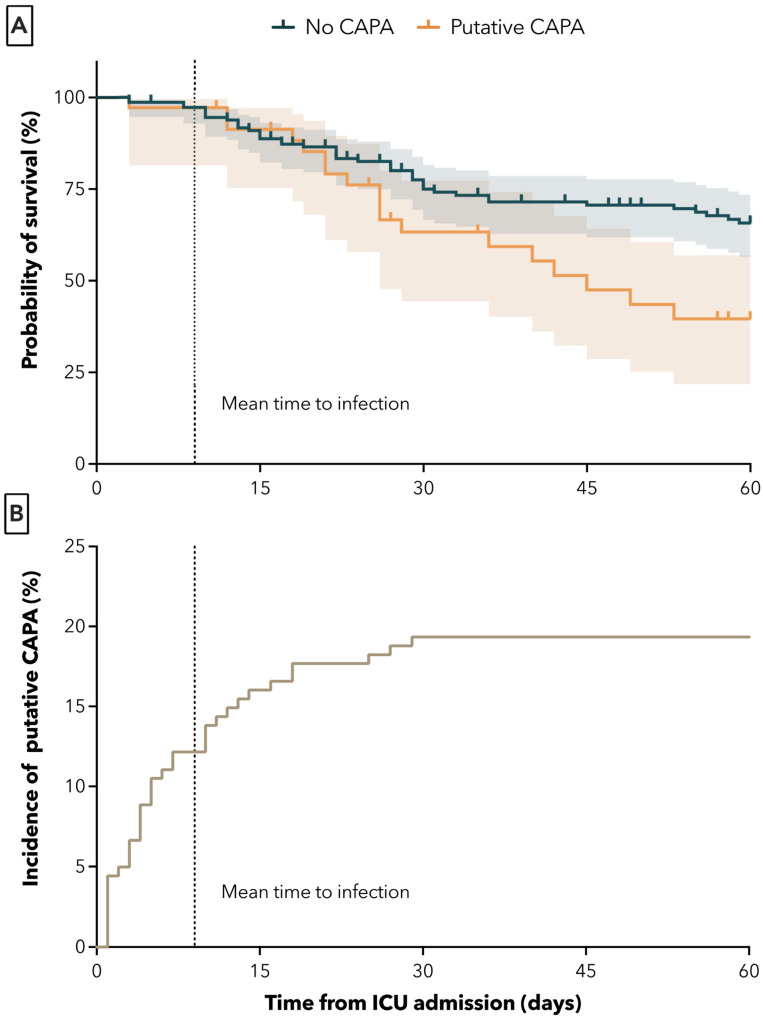
Survival probabilities of patients with and without putative aspergillosis in days after ICU admission (**A**) and mean time to first positive Aspergillus culture from BAL sample (**B**). Dotted lines represent mean time to infection.

The 60-day all-cause mortality from ICU admission was significantly higher in the CAPA group. Death at 60 days occurred in 18 of 35 (51.4%) of patients with CAPA and in 43 of 146 (29.5%) patients without CAPA (adjusted HR (95% CI) = 2.15 (1.20–3.86, *p* = 0.002). The unadjusted HR was 1.97 (95% CI 1.13–3.42), *p* = 0.01) (Figure 2A). The mean time from ICU admission to time of CAPA diagnosis was 9 ± 8 days (Figure 2B).

### 3.3. Pathogen Distribution

**Figure 3:**
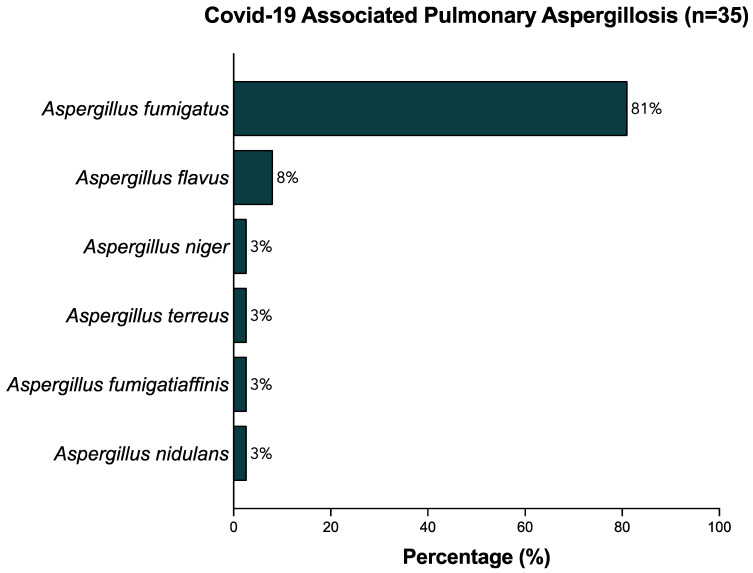
Relative frequencies (%) of strains identified in BAL samples.

The relative frequencies of identified aspergillus species in microbiological cultures of BAL samples are depicted in Figure 3. The most commonly identified *Aspergillus* species was *Aspergillus fumigatus*, which was found in 30 of 35 (85.6%) patients diagnosed with CAPA. *Aspergillus flavus* was found in three (8.6%) cases. One case each of *Aspergillus niger*, *Aspergillus terreus*, *Aspergillus fumigatiaffinis*, and *Aspergilllus nidulans* were identified (2.9%). One patient had a co-infection with three *Aspergillus* spp. (*A. Flavus*, *A. Fumigatus*, and *A. Niger*).

### 3.4. Antifungal Therapy

Table 2 provides an overview of the antifungal therapy used to treat CAPA. After the diagnosis of CAPA, treatment with voriconazole was initiated in 16 of 35 (45.7%) patients. Four patients (11.4%) received micafungin, three patients (8.6%) each received caspofungin or fluconazole, and one patient (2.8%) each received nystatin or isavuconazole. In 11 patients (31.4%), the dose was either increased or switched to another antifungal because symptoms did not improve.

## 4. Discussion

The present study provides vital insights into the prevalence and clinical outcomes of COVID-19-associated pulmonary aspergillosis in a population of critically ill patients. Our results demonstrate that CAPA is a significant complication, with high prevalence of 19% in patients who have undergone at least one BAL during ICU stays. The most commonly identified pathogen was *Aspergillus* fumigatus, which was found in 86% of cases. In line with the literature, the average time from ICU admission to the diagnosis of CAPA in our cohort was 9 days. This is consistent with the observation that invasive pulmonary aspergillosis (IPA) tends to occur later in the course of the illness in COVID-19 patients compared to influenza patients [13,18,19,20].

The diagnosis of aspergillosis can be challenging, as the symptoms are often nonspecific and may overlap with other respiratory infections [14]. Hence, previous studies examining the prevalence of CAPA in critically ill patients have yielded highly varying results, ranging from 2.5% to 35% [5,13,19,21,22,23,24]. Several criteria for the diagnosis of IPA have been proposed, but there is currently no international consensus regarding the use of any criteria for study purposes [17,25,26]. In addition, some studies rely on the use of galactomannan assays from blood or BAL samples for the diagnosis of IPA, which have only shown moderate sensitivity of about 70% in a large meta-analysis [15]. In addition, imaging techniques such as X-ray and CT scans may be useful in aiding the clinical diagnosis of aspergillosis but are not specific for the disease [13]. As a result, IPA is at risk of misdiagnosis when not strictly adhering to established diagnostic criteria, and the true prevalence of CAPA may be lower than previously reported, as suggested by a systematic review of autopsies [27,28].

Our study adds to the growing body of evidence demonstrating the significant impact of CAPA on mortality in COVID-19 patients. Our study found that patients with CAPA had significantly higher 60-day all-cause mortality compared to those without CAPA (51% vs. 30%). These results are consistent with the previous literature, which also observed higher mortality in COVID-19 patients with CAPA (44% to 55%) versus without CAPA (19% to 34%) [1,5,19,23,29]. It is important to note that most studies investigated shorter time periods, typically 30 days or less, which may not capture the full extent of the impact of CAPA on mortality. Here, we opted for 60-day all-cause mortality due to the late onset of CAPA in our cohort. The longer period of observation reveals diverging mortality curves after the mean time to infection, as shown in Figure 2. This highlights the importance of longer-term follow-up in patients with CAPA to fully understand the impact of this comorbidity on outcomes.

Although the clinical diagnosis of CAPA remains challenging, Gangneux et al. observed that a positive *Aspergillus* culture from respiratory specimens alone was associated with increased mortality, regardless of aspergillosis status [20]. Hence, due to high mortality in ICU patients with CAPA and delayed onset, days to weeks after ICU admission, patients may benefit from the early initiation of antifungal therapy as soon as *Aspergillus* is recovered [18]. However, the evidence supporting the use of antifungal therapy in the management of CAPA remains inconclusive. Hatzl et al. observed no improved survival after treating critically ill COVID-19 patients with antifungal prophylaxis at admission, despite a significant reduction in CAPA incidence [30]. These contradicting findings therefore warrant further research into the diagnosis, treatment, and outcomes of CAPA.

The use of corticosteroids to combat severe COVID-19 has become the standard of care after pivotal clinical trials [31]. However, previous studies have linked the use of immunosuppressants with the increased incidence of CAPA [11,12,32]. This raises concerns about the safety of corticosteroid use in patients with COVID-19, particularly those at high risk of developing CAPA. In our study, all patients with putative CAPA received corticosteroids at the time of diagnosis, as defined by the AspICU criteria. In addition, 73% of all patients received corticosteroids at the time of ICU admission. Although descriptively more patients without CAPA than with CAPA received immunosuppressants at ICU admission, there was no significant difference between the groups. This suggests that the use of corticosteroids in patients with COVID-19 may not be the sole contributing factor to the development of CAPA in our cohort.

The majority of patients with CAPA in our study received voriconazole as initial antifungal treatment (46%). In 31% of all patients, either the dose had to be increased or switched to another antimycotic because the symptoms did not improve. This highlights the challenges associated with managing CAPA in critically ill COVID-19 patients and the need for further research to identify more effective antifungal regimens.

Although limited to a single study center, the inclusion of a large number of patients from multiple ICUs allowed for a homogeneous, comprehensive assessment of the incidence and clinical outcomes of CAPA in this group of patients with comparable treatment practices and outcomes. Nevertheless, this design may limit the generalizability of our findings to other populations, as the patient population in our study center may not be representative of other regions or countries. Furthermore, the diagnosis of CAPA remains challenging, and the AspICU classification may not be fully sensitive or specific enough to diagnose this condition. Nevertheless, as discussed earlier, previous studies have demonstrated that positive cultures from respiratory specimens alone are associated with increased mortality, regardless of aspergillosis status. Hence, we opted for BAL cultures alone, as opposed to less sensitive galactomannan assays, for the identification of putative CAPA cases. Furthermore, previous studies have demonstrated increased mortality in critically ill COVID-19 patients with concomitant bacterial, viral, and fungal co-infections. The frequency of bacterial infections did not differ between patients with or without CAPA in our cohort. However, we were unable to obtain reliable data on other fungal or viral co-infections, which may have had an impact on clinical outcomes. Finally, the present study is limited by its retrospective design, which may be subject to bias or documentation errors in medical records.

## 5. Conclusions

In conclusion, the prevalence of putative pulmonary aspergillosis in critically ill COVID-19 patients was high (19%), with a mean time to diagnosis of 9 days after ICU admission. Patients with putative pulmonary aspergillosis had significantly higher mortality compared to those without CAPA. These results highlight the importance of the close surveillance of fungal co-infections in critically ill COVID-19 patients.

## Figures and Tables

**Table 1:** Baseline characteristics of the study cohort.

	Overall (n = 181)	No CAPA (n = 146)	Putative CAPA (n = 35)	*p*-Value
**Demographic characteristics**				
Female sex, n (%)	54 (29.8)	45 (30.8)	9 (25.7)	0.698
Age, mean (SD)	54.46 (13.03)	54.08 (13.56)	56.06 (10.58)	0.422
BMI, mean (SD)	30.73 (7.13)	30.86 (7.52)	30.17 (5.24)	0.609
**Length of hospital stay (days),** **mean (SD)**	36.73 (25.53)	35.92 (25.41)	43.28 (26.30)	0.250
**Antigen assays ^a^**				
Galactomannan index, median (IQR)	0.10 (0.06–0.23)	0.08 (0.06–0.15)	3.69 (0.16–7.04)	0.0001
Positive galactomannan index, n (%)	22 (16.4)	4 (3.7)	18 (66.67)	0.0001
β-D-Glucan (pg/mL), median (IQR)	74.83 (0.00–194.2)	72.59 (0.00–169.6)	142.2 (51.47–365.1)	0.02
Positive β-D-Glucan, n (%)	66 (47.1)	49 (43)	17 (65.4)	0.039
**Bacterial infection ^b^**	46 (25.4)	39 (26.7)	7 (20)	0.52
**Baseline comorbidities, n (%)**				
Diabetes	24 (13.3)	16 (11.0)	8 (22.9)	0.113
Obesity	84 (46.4)	74 (49.3)	10 (28.6)	0.03
Coronary artery disease	13 (7.2)	13 (8.9)	0 (0.0)	0.142
Chronic heart failure	4 (2.2)	2 (1.4)	2 (5.7)	0.352
Asthma	5 (2.8)	4 (2.7)	1 (2.9)	1.000
Chronic obstructive pulmonary disease	9 (5.0)	8 (5.5)	1 (2.9)	0.835
Hypertension	42 (23.2)	32 (21.9)	10 (28.6)	0.539
Chronic kidney disease	15 (8.3)	12 (8.2)	3 (8.6)	1.000
Skin disorder	16 (8.8)	15 (10.3)	1 (2.9)	0.291
Mental disorder	33 (18.2)	31 (21.2)	2 (5.7)	0.059
Neurologic disorder	20 (11.0)	19 (13.0)	1 (2.9)	0.155
Neoplasm	26 (14.4)	19 (13.0)	7 (20.0)	0.429
**Immunosuppressants at ICU admission**	133 (73.5)	110 (75.3)	22 (62.9)	0.135
**SARS-CoV-2 variant**				0.213
Wildtype	34 (18.8)	28 (19.2)	6 (17.1)	
B.1.1.7 (Alpha)	80 (44.2)	66 (45.2)	14 (40.0)	
B.1.617.2 (Delta)	54 (29.8)	39 (26.7)	15 (42.9)	
Unknown	13 (7.2)	13 (8.9)	0 (0.0)	

^**a**^ The galactomannan assay was available in 107 patients in the No-CAPA group and in 27 patients in the putative CAPA group. The β-D-Glucan assay was available in 114 patients in the No-CAPA group and in 26 patients in the putative CAPA group. ^**b**^ Samples were collected from respiratory tract and blood.

**Table 2:** Overview of baseline immunosuppressive medication, pathogen prevalence, time to infection, antifungal therapy, and outcome by patient. n/a refers to data not available in medical records; [-] indicates no use of second-line antifungal therapy.

Patient	Sex	Age (Years)	Immunosuppressants at ICU Admission	*Aspergillus* spp.	Time to Infection from ICU Admission (Days)	Initial Antifungal Therapy at Time of Infection	Second-Line Antifungal Therapy	Outcome
**1**	M	49	Dexamethasone 12 mg	Fumigatus	11	Voriconazole 200 mg	Voriconazole 400 mg	Recovered
**2**	M	35	Prednisolone 100 mg	Fumigatus	1	Caspofungin 70 mg	Voriconazole 400 mg	Recovered
**3**	F	46	Hydrocortisone 200 mg	Fumigatus	10	Voriconazole 300 mg	[-]	Recovered
**4**	M	50	None	Fumigatus	1	Micafungin 100 mg	Voriconazole 400 mg	Death
**5**	M	50	Dexamethasone 4 mg	Fumigatus	2	Voriconazole 400 mg	[-]	Death
**6**	M	49	None	Flavus	3	Voriconazole 200 mg	[-]	Recovered
**7**	M	59	None	Fumigatus	4	Voriconazole 200 mg	[-]	Death
**8**	M	46	Prednisolone 50 mg	Fumigatus	29	Fluconazole 800 mg	[-]	Recovered
**9**	M	27	Dexamethasone 40 mg	Flavus	1	Voriconazole 400 mg	[-]	Death
				Fumigatus	1			
				Niger	1			
**10**	M	64	Prednisolone 50 mg	Fumigatus	10	n/a	n/a	Death
**11**	M	45	None	Fumigatus	18	n/a	n/a	Death
**12**	M	56	Dexamethasone 10 mg	Fumigatus	4	Voriconazole 400 mg	[-]	Death
**13**	M	63	None	Fumigatus	1	Voriconazole 200 mg	[-]	Death
**14**	M	51	Dexamethasone 6 mg	Fumigatus	1	Voriconazole 400 mg	[-]	Death
**15**	M	67	Dexamethasone 8 mg	Fumigatus	4	Nystatin 1 mL	[-]	Recovered
**16**	F	55	Prednisolone 25 mg	Fumigatus	5	Voriconazole 300 mg	[-]	Recovered
**17**	F	61	None	Terreus	18	Voriconazole 400 mg	[-]	Recovered
**18**	M	57	Dexamethasone 8 mg	Fumigatus	1	Voriconazole 200 mg	[-]	Death
**19**	M	56	Dexamethasone 6 mg	Fumigatus	5	Fluconazole 600 mg	Voriconazole 200 mg	Death
**20**	M	61	None	Fumigatus	3	Isavuconazole 200 mg	[-]	Death
**21**	F	69	None	Fumigatus	13	n/a	n/a	Death
**22**	M	70	Dexamethasone 10 mg	Fumiigatiiaffinis	12	Micafungin 100 mg	Voriconazole 400 mg	Death
**23**	M	70	Dexamethasone 6 mg	Nidulans	10	n/a	n/a	Death
**24**	M	48	Dexamethasone 6 mg	Fumigatus	1	Voriconazole 200 mg	Voriconazole 300 mg	Recovered
**25**	F	62	None	Fumigatus	1	Voriconazole 200 mg	Voriconazole 200 mg Nystatin 0.5 mL	Recovered
**26**	M	60	None	Flavus	27	n/a	n/a	Recovered
**27**	M	54	Dexamethasone 10 mg Anakinra 600 mg	Fumigatus	7	Fluconazole 800 mg	Voriconazole 200 mg	Death
**28**	M	58	Hydrocortisone 200 mg	Fumigatus	6	Caspofungin 70 mg	Amphotericin B 600 mg	Recovered
**29**	F	69	None	Fumigatus	16	Caspofungin 50 mg	n/a	Death
**30**	F	79	Dexamethasone 4 mg	Fumigatus	5	n/a	n/a	Death
**31**	F	51	Dexamethasone 4 mg	Fumigatus	3	Voriconazole 200 mg	Voriconazole 400 mg	Recovered
**32**	M	54	None	Fumigatus	4	Micafungin 100 mg	n/a	Recovered
**33**	M	63	Prednisolone 250 mg	Fumigatus	25	n/a	n/a	Death
**34**	M	43	None	Fumigatus	14	Micafungin 100 mg	Micafungin 100 mg Amphotericin B 10 mg inhal.	Recovered
**35**	F	65	Prednisolone 500 mg	Fumigatus	7	Voriconazole 300 mg	[-]	Death

## Data Availability

The data and materials that support the findings of this study are available from the corresponding author upon the reasonable request.
